# Contagious Animal Diseases in Western New York

**Published:** 1884-10

**Authors:** Edward Crundall

**Affiliations:** Geneva, N. Y.


### Art. XXIV.—CONTAGIOUS ANIMAL DISEASES IN WESTERN NEW YORK.

BY EDWARD CRUNDALL, V.S.

In responding to your invitation to furnish for the Journal an account of the prevalence of contagious diseases of animals in my district, I may say that, excepting tuberculosis in cattle and glanders in horses, contagious diseases have not prevailed to any particular extent in my neighborhood for some time past.

We have had a few cases of influenza in horses, assuming a very mild form, and not followed in any case by serious sequelæ. It did not appear to be contagious. In a stable of a dozen or more horses, only one or two were affected, and in several instances, where only two horses were kept, only one had the disease.

I have had one case of scarlatina this Spring in a bay gelding seven years old; it presented all the features of an ordinary case of influenza, with angina and catarrh, for the first few days; this was followed by a marked elevation of temperature (106° Fahr.), œdema and stiffness of the limbs, swollen condition of the eyelids, a few elevated patches about the head and nose, and to a slight extent, over the general surface of the body, with the characteristic bright scarlet spots on the inner surface of the lips and mucous membrane covering the nasal septum. With the administration of febrifuges and salines, followed by mild stimulants and tonics, good nursing, etc., the animal became convalescent in about three weeks. I tested the urine for albumen several times, but only on two occasions did I find any, and then it was quite insignificant in amount; the albumen appeared after the sore throat, cough, and catarrh had entirely subsided, and during a sudden increase in the œdema; it rapidly disappeared under tonic treatment.

There is nothing remarkable about this case, and I should not have noticed it at all, but that from the nasal discharge or saliva of the horse I became inoculated through the medium of a scratch on the back of my hand; and the train of symptoms which followed were so entirely different from anything I had ever experienced before from former inoculations with diseased products, and also seemed to my mind to militate, at least in a measure, against the identity of human and equine scarlatina. The following are the symptoms which presented: A day or two after the inoculation, the scratch on the back of the right hand where the virus entered became very much inflamed and swollen, and had the appearance of a large carbuncle; the swelling and inflammation extended all over the back portion of the hand as far forwards as the middle of the fingers, and backwards to nearly the middle of the forearm; it was only slightly painful, but intensely pruritic; the axillary glands became swollen and very painful in comparison with the local manifestations; the fever and constitutional disturbance was but slight; the parts remained in *statu quo* for three or four days, when other boils commenced to form, one on the forehead and other two on the back of the neck; not caring to have them maturate in these locations, I applied belladonna to them, and took calcium sulphide with quinine and stimulants internally; this most effectually prevented it. In the meantime the carbuncle at the point of inoculation had grown considerably larger, and presented a very indolent appearance, containing a large slough; the parts around it became considerably diminished in size, but the pruritis continued, in spite of local sedatives, almost unbearable. With continued poulticing, the slough separated in a few days more, being the ninth day after the inoculation; cicatrization went on favorably, the pruritis gradually subsided, and was followed by patches of eczema in different parts of the body; this yielded readily to treatment, and convalescence was fully established in about three weeks.

I submit this short and very imperfect history of a somewhat exceptional case to the readers of the JOURNAL, without making any remarks on it, or attempting to speculate on the nature or causes of scarlatina equina, excepting that I fail to recognize any similarity between the human and equine disease, and regard it as a scarlatina peculiar to horses, and not transmissible to man or other animals, or capable of being spread by either contagion or infection to man or animals. In fact, my experience of scarlatina in horses accords perfectly with what Prof. Williams teaches in his lectures and has written in his valuable work.

GLANDERS.

Owing to the unrestricted traffic going on in glandered horses by low, unprincipled dealers, this disease is of quite frequent occurrence. The law in this State, at least in my district, is entirely inoperative on account of the lack of proper persons whose duty it should be to put its machinery in motion; "it being every one's business, no one attends to it." It is by no means uncommon for dealers to knowingly trade off glandered horses, resorting to various devices to accomplish their object and deceive the unwary, thus scattering broadcast the seeds of this virulent and loathsome disease.

A few months ago I was requested by one of these gentry to visit two horses said by him to be suffering from what he called the epizootic. I found both of them presenting well-marked symptoms of glanders and farcy. Upon inquiring into the history of the cases, to ascertain if possible where the disease originated, the man informed me that he had some few weeks back disposed of a team of black horses which he traded for, that both had a discharge from the nose, and, to use his own expression, hard bunches under the chops, and he got them cheap on that account; he kept them for some time, endeavoring to stop the discharge and get them in swapping condition; but failing in this, he let them go as they were, to a member of the same fraternity living some distance from him. The horses, which I found glandered, had for several weeks occupied the same stalls as the black horses, which were undoubtedly glandered also, and they must have contracted the disease from contact with the diseased products still remaining in the stalls,

As they were both healthy when admitted, I informed him of the serious nature of glanders, the danger of transmission to man, etc., and posted him on the law relating to it, and also advised him to kill them both, and thoroughly disinfect his stalls before placing other horses in them. All this he promised to do at once; but, however, notwithstanding his good promises, he failed to kill the horses, although he removed them from his stables and sent them to a secluded place in the country. I afterwards learned that they both received treatment for some time at the hands of a woman, "the widow of a deceased horse doctor," who declared that her husband never failed to cure glanders; and that one of them died, and the other was destroyed, but not until repeated efforts had been made to dispose of them. Each of these horses had frequently been watered at the village watering-trough, as it was this man's custom to water all his horses there daily.

About two months afterwards another horse, owned a short distance out of the village, became glandered. This animal had been repeatedly watered at the same trough, and he probably contracted the disease there, although there are no means of proving it; yet, in the absence of proof to the contrary, it is fair to presume he did.

So much for the blessings conferred by public watering-troughs.

CATTLE.

The disease most prevalent in this district amongst cattle is tuberculosis; other diseases of cattle in this neighborhood are quite insignificant when compared with this bovine scourge. I have no hesitation in saying, from careful investigation, that fully thirty per cent. of the grade cattle in the counties of Seneca and Ontario are affected with tuberculosis. In the pure-bred herds the percentage used to be much higher; but of late my experience with these latter has been very limited, as, owing to the losses caused by the disease, nearly all the herds originally kept here have been broken up and discontinued. You can pick one or two tuberculous cows out of every farmer's barnyard throughout the country; they (the farmers) very seldom lose an animal with it, as they do not keep them long enough for the acute stage to set in. Whenever a cow becomes nympho-maniac and fails to become pregnant, she is fattened and sold to the butcher without delay, as they know full well what will follow, from former experience; or whenever signs of ill-health are apparent, from whatever cause, in an animal, after a few doses of medicine, and a slitting of the tail and boring of the horns for the so-called hollow horn by some brute quack, if it then fails to recuperate at once, it is sold to a second or third-rate butcher for whatever it will bring. This conscientious tradesman exposes the meat for sale as "choice meat." Animals that are too poor to make decent-looking beef are converted into "fresh-cured bologna sausages."

All the dairymen, with scarcely an exception, are selling milk from tuberculous cows.

Although they are not generally aware of it, as long as a cow continues to give milk, no matter what her condition may be, it is milked into the pail and mixed with the milk from healthy cows, and sold to the unsuspecting consumer. Many a mother who consoles herself that her infant is getting the best of milk because it all comes, "as she supposes, at least," from one cow, is unknowingly feeding it poison from poisoned milk reservoirs, which at some future time will be the means of committing it to a premature grave, and its death will be attributed to cholera infantum or something else, without even a suspicion of intestinal tuberculosis from drinking—"yes, living upon"—tuberculous milk during the first months of its existence.

If the time at my disposal would permit, I could give overwhelming evidence of the communicability of tuberculosis from one animal to another from being stabled together, and also of hereditary transmission. However, two illustrations must for the present suffice:

A client of mine, who keeps a few very well-bred short-horn cattle, has had good success for a number of years, and been peculiarly fortunate in not having had any disease of importance among them until about three years ago, when it happened that, having occasion to change his bull, he went to Canada and bought a two-year-old short-horn bull of the Booth family. Shortly after this new purchase arrived at the farm, he was noticed to cough occasionally. This, however, was attributed to catching cold, and no importance was attached to it. He served that season nearly all of the cows on the farm—about a dozen in all—and also several others for neighboring farmers. During the following winter a two-year-old heifer, which stood in a stall in quite close proximity to the new bull, was observed to cough occasionally, especially when she went from the warm byre out into the barnyard, and her general condition was not as good as usual. Little notice was taken of this either—it was also attributed to local causes—until, " to make a long story short," three or four others became affected in the same way. The ensuing spring three of these cows were nympho-maniac, and I was requested to dilate their *os uteri*, with the hope that they might become pregnant. As I had predicted, however, this failed, after repeated dilatations at the owner's urgent request. They were then fattened and sold, and I had the opportunity of inspecting the carcase of one of them in the slaughter-house. Miliary tubercles were present in nearly all the organs, and had in different parts become aggregated together, forming large masses of different sizes ; degenerative processes had advanced in varying degrees ; the lungs had increased considerably, both in size and weight ; the parenchyma had broken down in places, and formed cavities, etc., etc. Another cow aborted at the fifth month of pregnancy ; acute tuberculosis followed, and she was killed. Three of the calves, the get of this bull, became tuberculous to my knowledge ; how many more, I am unable to say. The bull's cough grew worse, and all the symptoms of the disease increased ; he was unable to hold his condition, and became impotent ; he was sold to a bologna sausage manufacturer, who converted him into bolognas. My client is now satisfied that the cause of all this trouble was the Canada bull. This small herd was remarkable for its healthy condition previous to the admission of the fresh bull.

Early in the summer of 1882, while visiting a lame horse at a farm in connection with a well known institution situated in the western part of this State, my attention was casually called by the steward to a cow in one of the pasture fields. This animal exhibited the well known symptoms of tuberculosis in the second stage. As I had but a few minutes to catch the train on which I was to return home, and also as I had not been requested to examine any other animals in the herd, I satisfied myself by simply inquiring if any of the others were affected in a similar manner. The herdsman informed me that one other seemed to be similarly affected, but that it was not as bad as the one I had seen. I informed the steward that this cow was tuberculous, and that I regarded it as a contagious disease, and advised him to remove these two animals from the rest of the herd, keep close watch over the others, and in case any more became diseased, to at once remove them, as I feared that unless vigorous measures were taken to stamp it out by separating the diseased from the healthy, etc., it might be communicated to nearly every animal in the herd. This gentleman was very much astonished at my serious apprehensions, as he had never before heard of tuberculosis in cattle; and, "be it said to his credit," was inclined to act upon my suggestions at once by having the two already diseased killed; but upon communicating with the director of the institution, who is a physician, his fears were at once allayed, and his ardor to stamp out the disease by proper sanitation effectually cooled. He was led to the belief that the disease was not contagious, that there was no necessity for taking any extraordinary measures, and that doubtless it would soon subside, etc. This resulted in my advice being utterly ignored. The disease progressed "without let or hindrance," until it began to assume alarming proportions. During the last winter the steward sent for me to examine the herd, with a view to ascertain how many of them were diseased, and to institute means to prevent the destruction of the whole, numbering about 175 grade cattle. I being absent from home, another veterinary surgeon was consulted; he confirmed my suspicions, and gave the same advice that I had given two years before, which is now being acted upon, "and which reminds one of locking the stable door after the horse is gone."

I am informed by the butcher who killed and dressed the fat cattle for the institution, that nearly every other animal dressed by him showed traces of the disease, and also that out of eleven that died he made autopsies on nine, and found each one tuberculous. He informs me also that the offal from the diseased cattle was all fed to the hogs, and that they also have become diseased. He found in them tuberculous deposits, "similar, he says, to those found by him in the cattle," in the lungs and abdominal viscera, and also large metastatic abscesses in the muscles. I may state that the butcher is quite an intelligent man, and he described tolerably well the morbid appearance which presented, at least so as to satisfy me of the probable correctness of his statement.

Actinomycosis in cattle is quite a frequent disease here, and notwithstanding the medical record implies that it was first discovered in American cattle by Dr. Bellfield, and that the veterinary profession knew nothing of it until their attention was called to it by him, the fact is, that the disease was at once recognized by the profession of this country and Canada shortly after Bollinger's discovery became known to them through the medium of the journals.

Speaking for myself, I can state that being induced, through accounts of the disease "in the journals," to examine these growths microscopically during the summer of 1882, I at once recognized the mycosis, and became satisfied of the identity of Bollinger's actinomycosis and the so-called osteo sarcoma.

Comparatively few of these cases are submitted to veterinarians for treatment, especially among the ordinary grade cattle which obtain in my district; however, I occasionally have a case of it to treat, and usually get good results from excision of the diseased parts, and after dressing with idoform. I have not met with any infectious or contagious diseases among sheep, with the exception of tuberculosis and scab. Quite a large percentage are affected with the former, and the latter is never absent, owing to the unrestricted movements of scabby flocks. In hogs, judging from the numbers of people I know that have played the part of host to teniæ solium, measles must be common amongst them, although I have only found them twice in pork ; but this is owing to lack of opportunity.

I am unable to judge of the prevalence of trichinæ, as I regret to say that so far neither opportunity nor time have permitted me to make any examinations.

In dogs, excepting the usual amount of parasitism, there is no disease. I have not seen a case of distemper for some time.

Large numbers of young cats die from the invasions of ascaris mystax.

In closing this hastily-prepared and very imperfect sketch, I may state that in a country where there is an abundance of healthy animal food, it is to be regretted that such an extensive trade in diseased animals, meat, and impure milk should be constantly going on; it is certainly opposed to either agricultural or national prosperity, and must be a potent cause in the production of human diseases and of human mortality.

If a competent system of meat and dairy inspection were instituted by the Government or local authorities, and carried out by educated veterinarians, graduates of accredited colleges, it would certainly most effectually check this now rapidly increasing trade, and confer a lasting boon on the country.

GENEVA, N. Y., *Midsummer*, 1884.

